# Is Preterm Birth a Risk Factor for Subsequent Autism Spectrum Disorder and Attention Deficit Hyperactivity Disorder in Children with Febrile Seizure?—A Retrospective Study

**DOI:** 10.3390/life11080854

**Published:** 2021-08-20

**Authors:** Chien-Heng Lin, Wei-De Lin, I-Ching Chou, Inn-Chi Lee, Syuan-Yu Hong

**Affiliations:** 1 Division of Pediatrics Pulmonology, China Medical University Children’s Hospital, Taichung 404327, Taiwan; lch227@ms39.hinet.net; 2 Department of Biomedical Imaging and Radiological Science, College of Medicine, China Medical University, Taichung 404327, Taiwan; 3 Department of Medical Research, China Medical University Hospital, Taichung 404327, Taiwan; weide@mail.cmuh.org.tw; 4 Division of Pediatrics Neurology, China Medical University Children’s Hospital, Taichung 404327, Taiwan; iching@mail.cmu.edu.tw; 5 Department of Pediatrics, Chung Shan Medical University Hospital and Institute of Medicine, School of Medicine, Chung Shan Medical University, Taichung 402306, Taiwan; y610@mercury.csmu.edu.tw; 6 Department of Medicine, School of Medicine, China Medical University, Taichung 404328, Taiwan; 7 Institute of Biomedicine, School of Medicine, China Medical University, Taichung 404328, Taiwan

**Keywords:** epilepsy, preterm, attention deficit hyperactivity disorder, autism spectrum disorder, neurodevelopmental disorders

## Abstract

Febrile seizure (FS) is the most prevalent childhood seizure; it is significantly related to subsequent epilepsy and has possible links to childhood neurodevelopmental disorders. Separately, premature births are believed to increase the risk of attention deficit hyperactivity disorder (ADHD) and autism spectrum disorder (ASD). Therefore, this study investigated whether preterm birth is a risk factor for subsequent epilepsy, ASD, and ADHD in children with FS. We retrospectively collected data for children aged < 5 years with FS from 1 January 2005, to 31 December 2013. We divided these children into two groups—the premature birth group and the full-term group—and compared their incidence rates of epilepsy, ASD and ADHD. The data of 426 patients with history of febrile convulsion were retrospectively collected. The premature birth group (FS+/preterm+) had 108 patients and the full-term group (FS+/preterm−) had 318 patients. The overall epilepsy risk in the FS+/preterm+ group was higher than in the FS+/preterm− group (odds ratio [OR], 2.52; 95% confidence interval [CI], 1.14–5.58; *p* = 0.02). The overall risk of ADHD in the FS+/preterm+ group was higher than that in the FS+/preterm− group (OR, 6.41; 95% CI, 3.39–12.09; *p* = 0.0001). In addition, children with FS+/preterm+ had 16.9 times (95% CI, 4.79–59.7; *p* = 0.0001) higher odds of having ASD compared with those with FS+/preterm−. Preterm birth may be a risk factor for subsequent epilepsy, ASD and ADHD in children with FS.

## 1. Introduction

Autism spectrum disorder (ASD) and attention deficit hyperactivity disorder (ADHD) are two common neurodevelopmental disorders (NDDs) in children. ASD, which commonly manifests in the first years of life, is a disease with multifactorial etiology and typically manifests as social and communication impairments as well as stereotyped behaviors, whereas ADHD is characterized by inattention, hyperactivity and impulsivity. These disorders are thought to have a strong heritable component and identified risk factors include extremes of maternal or parental age, mothers with chemical intolerances, low socioeconomic status, prenatal exposure to smoking and alcohol and epilepsy [1,2,3,4,5,6,7].

Febrile seizure (FS), which has an onset age mostly between 14 and 18 months, is the most common childhood seizure, affecting 2–5% of children aged < 5 years [8]. Although FS is an inherently benign process, it can recur in approximately 30% of cases, 2% of which may develop epilepsy [9]. Certain mental and neurodevelopmental consequences of FS have been identified, particularly ASD and ADHD [10,11,12,13]. Furthermore, some authors have reported that there is a strong association between epilepsy, FS and subsequent development of ADHD, especially in areas with high levels of urbanization [10,13]. Preterm birth is a major cause of death worldwide and can lead to major health problems. In Taiwan, approximately 200,000 infants are born annually, and the preterm birth rate is estimated to be 8–10%. Infants born prematurely are at risk of major and minor defects, such as cerebral palsy, cognitive and speech delays, motor and visual deficits, psychosocial and behavioral disorders and dysfunction at school [14,15]. Therefore, studies have mentioned preterm birth as a risk factor for subsequent ADHD, especially in girls [15,16]. 

In this small, but clinically significant study, based on the abovementioned considerations, we sought to examine whether preterm birth acts as a prognostic factor for subsequent neurodevelopmental sequelae in children with FS. Our hypothesis was that preterm birth is a risk factor for not only epilepsy but also subsequent ASD and ADHD in children with FS.

## 2. Methods and Approach

### Patient Population

The study protocol was approved by the Ethics Review Board of the China Medical University Ethics Committee (CMUH108-REC1-023). In this retrospective study, we collected data from children aged 0–5 years who were born preterm and full-term and had been diagnosed with FS between 1 January 2005 and 31 December 2013. All subjects in the study (FS+/preterm+ and FS+/preterm−), were born in the China Medical University Children’s Hospital and its three branches and their birth medical records were complete and detailed. Preterm birth in the present study was defined as any birth before 37 completed weeks of gestation. The diagnosis of FS was made by child neurologists in accordance with the following generally accepted criteria: (1) convulsions associated with an elevated temperature greater than 38 °C; (2) child aged older than 6 months and younger than 5 years; (3) absence of central nervous system infection or inflammation; (4) absence of acute systemic metabolic abnormality that may produce convulsions; and (5) no history of afebrile seizures [4].

**Figure 1:**
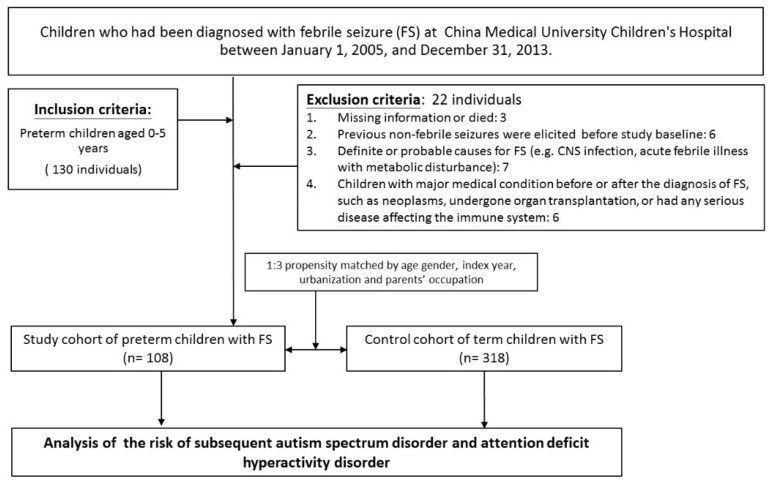
Study population and flowchart.

All patients included in the study were followed up until the end of follow-up (31 December 2017). We followed up patients by reviewing their medical records and contacting their families via telephone or e-mail quarterly since the beginning of 2014, to investigate whether a first diagnosis of epilepsy, ASD, or ADHD after FS was confirmed. Since we included patients who were sure to be contacted, there were three lost cases in the follow-up (Figure 1).

Subsequently, we comprehensively reviewed the respective medical records toward the end of 2017. The exclusion criteria for study participants were as followings: those individuals who had died during follow-up, those that had been diagnosed with neoplasms, had undergone organ transplantation, or had any serious disease affecting the immune system (e.g., systemic lupus erythematosus or aplastic anemia) before or after FS. The children enrolled in the study were relatively healthy; that is, they had no epilepsy or neurological, metabolic, autoimmune, or any known congenital disorders before the onset of FS. The final study population comprised 108 patients (FS+/preterm+).

In addition, each patient in the FS+/preterm+ cohort was 1:3 propensity matched by age, sex, index year, urbanization to form a control group of 318 full-term children with FS (FS+/preterm−). Epilepsy was defined as two unprovoked seizures more than 24 h apart as diagnosed by a pediatric neurologist. Patients who met the relevant diagnostic criteria in the Fourth and Fifth Editions of the Diagnostic and Statistical Manual of Mental Disorders (DSM-4, DSM-5) were diagnosed with ASD and ADHD and their diagnoses were made by a pediatric psychiatrist or pediatric neurologist in an inpatient or outpatient setting in the China Medical University Children’s Hospital between 1 January 2005 and December 31, 2017. Confounding factors were sex, gestational age, birth body weight, first FS duration and onset age, brain condition within 6 months of age and number of FSs (Table 1).

## 3. Statistical Analysis

Categorical variables between groups were analyzed using χ2 tests. Furthermore, we calculated the incidence density rates of epilepsy, ADHD and ASD in both groups (which were subdivided into different age intervals and subdivided by number of FSs). In addition to term and preterm, other confounders, such as sex, gestational age, birth body weight, first FS duration and onset age, brain condition within 6 months of age and number of FSs were analyzed and controlled by propensity matching and stratification in the process of logistic regression model [17]. However, the approach of analysis of covariance is a statistical linear model with a continuous outcome variable and two or more predictor variables where at least one is continuous and at least one is categorical, which is not applicable in this analysis. We used two sample t-tests for gestational age and birth body weight, first FS duration and onset age, number of FSs and chi-square tests for sex, urbanization, brain condition within 6 months of age and parent occupation to compare the difference between FS+/preterm+ and FS+/preterm− cohorts (Table 1). Using a logistic regression, we estimated the odds ratios (ORs) and 95% confidence intervals (CIs) of epilepsy, ADHD and ASD in the FS+/preterm+ group relative to the FS+/preterm− group. All statistical analyses were performed using PASW Statistics version 18.0 (SPSS Inc., Chicago, IL, United States). In addition, for all statistical analyses executed, we considered the two-tailed *p* < 0.05 to be statistically significant.

## 4. Results

### Data Analysis

**Figure 2:**
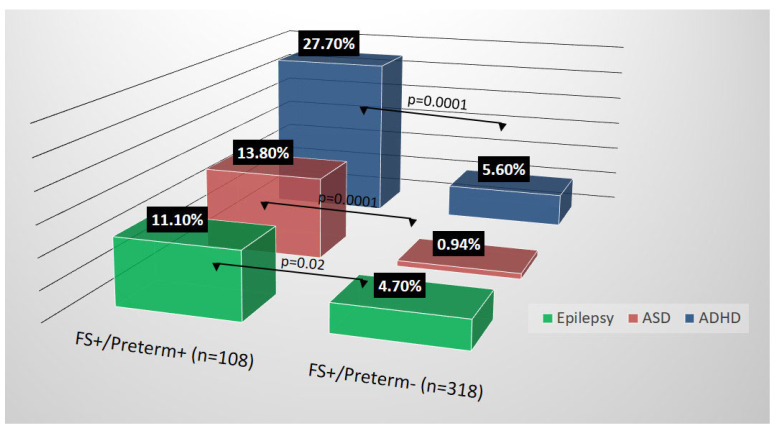
Incidence rates of epilepsy, autism spectrum disorders (ASD) and attention deficit hyperactivity disorder (ADHD) associated with children with febrile seizure in this study.

In total, 426 children who had been diagnosed with FS between 1 January 2005 and 31 December 2013 were enrolled into this study. Table 1 presents the participants demographic factors. The participants mean age was 2.3 years (standard deviation = 1.14) in FS+/preterm+ group and 2.19 years (standard deviation = 1.10) in FS+/preterm− group. The proportion of boys was higher than that of girls in both groups. Related brain imaging abnormalities in the study group were classified as negative (*n* = 45, 41.6%), intraventricular hemorrhage (*n* = 45, 41.6%), hydrocephalus or ventriculomegaly (*n* = 6, 5.5%), white matter damage or periventricular leukomalacia (*n* = 12, 11.1%) and others (*n* = 6, 5.5%). Figure 2 illustrates the NDDs associated with children with FS according to their incidence rates. 

Table 2 compares the FS+/preterm+ and FS+/preterm− groups in terms of their incidence rates and relative risks of epilepsy, ADHD and ASD. The overall epilepsy risk in the FS+/preterm+ group was higher than that in the FS+/preterm− group (OR, 2.52; 95% CI, 1.14–5.58; *p* = 0.02). The overall risk of ADHD in the FS+/preterm+ group was higher than that in the FS+/preterm− group (OR, 6.41; 95% CI, 3.39–12.09; *p* = 0.0001). Furthermore, the risk of ADHD among patients in the FS+/preterm+ group who experienced FS without recurrence (OR, 17.5, 95% CI, 6.95–44.0; *p* = 0.0001) and whose age of FS onset ranged between 1 and 5 years were even higher than those in the FS+/preterm− group (OR, 7.88; 95% CI, 3.70–16.7; *p* = 0.0001).

In addition, children in the FS+/preterm+ group had 16.9 times (95% CI, 4.79–59.7; *p* = 0.0001) higher odds of having ASD compared with those in the FS+/preterm− group, which was the same in each stratification subgroup. Among other things, patients in the FS+/preterm+ group who had experienced one episode of FS and whose age at FS onset was greater than 1 year had an even higher risk of ASD than those in the FS+/preterm− group (OR, 64.0, 95% CI, 7.35–557, *p* = 0.0001; OR, 20.27, 95% CI, 4.4–92.8; *p* = 0.0001)

Table 3 further compares the possible confounders in children within the FS+/preterm+ group for the development of ADHD and ASD. No significant differences were found in terms of sex, gestational age, brain condition, number of FSs, first FS duration and whether the children were small for their gestational age.

## 5. Discussion

Significant prognostic markers for subsequent epilepsy in FS were confirmed, including a family history of epilepsy, complex FS, focal FS, short fever duration before seizure, late onset of FS (age > 5 years) and multiple FS recurrences. Multiple FSs increased the risk of epilepsy [18]. Neurological sequelae, including new neurological deficits, intellectual impairment and behavioral disorders, are rare following FS. Studies have speculated a possible link between FS and ASD or other NDDs in children; however, they have lacked relevant predictors [10,12,13]. In this study, we attempted to indicate a possible linkage between FS and other NDDs as well as highlight that preterm labor may be a risk factor in the development of epilepsy as well as ASD and ADHD. Through this retrospective study, we demonstrated that preterm children with FS had a significantly increased incidence rate of subsequent epilepsy, ADHD and ASD compared with full-term children with FS. Furthermore, our results showed that the trend of preterm birth acting as a risk factor for FS to the development of ASD and ADHD is much more evident than that of epilepsy. 

Although not the main purpose of this study, our results revealed that 11.10% of preterm patients with FS developed epilepsy, whereas 4.7% of full-term patients with FS developed epilepsy, both of which were higher than figures previously reported in the literature. Moreover, we found that preterm birth might be a risk factor in combination with FS for the development of childhood epilepsy. However, there was no statistical significance in which we stratified according to the onset age and recurrence of FS. We believe that the formation of epilepsy is much more complicated and so individual risk factors selected in the present study cannot exist independently, that is, there is an interaction between various potential risk factors [18,19,20]. As we know, children with simple FS exhibit an increased association with the development of epilepsy [21] and a slightly higher risk of subsequent epilepsy (approximately 1%), compared with that in the general population (approximately 0.5%) [22]. The risk of future epilepsy in children with complex FS is approximately 4–6% depending on the number of complex features [22]. However, we did not differentiate between simple and complex FS in our study because parent descriptions of children’s seizures documented in medical records were mostly unreliable. Hence, further research is required to identify whether preterm birth poses a higher risk than complex FS in the development of epilepsy or other NDDs.

Studies have noted the relationship between FSs and ADHD, with some reporting FS to be a risk factor for subsequent ADHD [10,12,13]. Ku et al. reported that FS increases the risk of subsequent ADHD in areas with high levels of urbanization, as well as that recurrent FS increased the cumulative incidence of ADHD [12]. Salehi et al. demonstrated that hyperactivity had a significant relationship with FS among male patients [23]. However, whether FSs increase the risk of ADHD is a long issue of debate since conventional concepts believed simple FS had little effect on the adverse effects between behavior, academic performance and neurocognitive attention. Interestingly, only a few studies mentioned on the potential risk factors, such as age of the 1st FS onset, recurrence of FS, prematurity or not and so on [13,24]. On the other hand, despite years of controversy regarding the relationship between ASD and epilepsy, no definite consensus has been reached to establish the nature of the relationship [25]. Some population-based studies have identified a bidirectional relationship in which not only patients with epilepsy are at increased risk of suffering from certain neurological and psychiatric disorders (e.g., ASD, depression, anxiety disorders, ADHD and psychosis), but also patients with such conditions are at increased risk of epilepsy [26]. Other studies have suggested shared mechanisms between epilepsy and ASD [27,28,29]. Gillberg et al., conducted a leading study regarding FS and its association with ASD [10]. Following Gillberg et al., our present study further identified higher incidence rates of ADHD and ASD in preterm children with FS. 

Our results, on the contrary, revealed some differences than before. After taking gestational age into account, we found that FS onset above 1 year of age might significantly increase the risk of ASD and ADHD, but the puzzling thing is that children without recurrence of FS had an even higher risk of developing ASD and ADHD. The above phenomenon did not appear when considering epilepsy. Although we have obtained new findings from our observational data, there is no reasonable explanations for them yet. Therefore, further studies to determine the role of prematurity in the development of ADHD and ASD across the different stages of the developing brain will be essential to elucidate our findings [30,31]. 

Preterm birth is the leading cause of neurobehavioral impairment and disability, causing a large economic burden [32,33]. Studies have found a higher prevalence of prematurity in children with ADHD or ASD, as well as that preterm birth increased the risk of subsequent ADHD or ASD [16,34,35,36,37]. The shorter the gestation age of those infants, the higher their risk of ASD [37]. However, when we compared the possible confounders within the FS+/preterm+ group in terms of the development of ADHD and ASD, we did not notice any significant difference between them (Table 3). Such a result was incongruous with previous findings [35,37]; thus, future studies may employ larger study samples and more in-depth study designs.

Pathophysiologically speaking, myelination processes and brain development are interrupted in prematurely born children and the connectivity between brain regions could be disrupted as well; this may explain the occurrence of NDDs and the emotional/behavioral problems in this population [38]. Despite the benign nature of FS, a genetic predisposition to the condition has been recognized; studies have disclosed that genetic variants in loci containing sodium channel genes (e.g., SCN1A and SCN1B among others) [39], could impede human brain development and are probably related not only to childhood epilepsy, but also to intellectual disability and ASD [40,41,42]. In other words, there might be a genetic variant that predisposes children to febrile seizures or epilepsy with febrile seizures and is also associated with abnormal brain development and subsequent epilepsy, intellectual disability and ASD [43,44]. Thus, we believe that preterm children with repeated FSs are at risk of epilepsy and NDDs and more studies are necessary to determine whether there is the same genetic variant between them.

This study had some limitations. First, some confounding factors may have affected the results, such as the mother’s medical condition during pregnancy, socioeconomic status, pharmacological treatment of children with preterm birth, malnutrition and environmental factors. Second, our sample size was relatively small and thus a population-based cohort study should be designed to prove our findings. Third, we did not perform an analysis of seizure semiology because of restrictions on the study design. Fourth, preterm children are monitored much more closely for developmental delay and therefore are more likely to be diagnosed having ASD or ADHD.

A large prospective cohort longitudinal study that investigates whether the incidence rates of ASD and ADHD are significantly different between preterm and control groups may help to confirm our findings. Furthermore, additional research that focuses on neuroimaging, genetic susceptibility, iron homeostasis, or proinflammatory cytokines may identify potential mechanisms.

## 6. Conclusions

This study investigated whether preterm birth is a risk factor for subsequent epilepsy, ASD and ADHD in children with FS. It is crucial for pediatricians to identify the early symptoms of ADHD or ASD in children with a history of FS—especially those born prematurely—to initiate proper assessment and treatment. Additional patient data and more in-depth studies are warranted to elicit potential cofounders to clarify the influence of preterm labor on FS and its association with ADHD and ASD. 

## Figures and Tables

**Table 1:** Demographic and Clinical Characteristics of Preterm Children with Febrile Seizure (FS) and a Comparison with Full-Term Children with FS.

Demographic and Clinical Characteristics	Group		
	FS+/Preterm+ (*n* = 108)	FS+/Preterm− (*n* = 318)	*p*
Mean 1st FS onset age (yrs) (SD) *	2.30 (1.14)	2.19 (1.10)	0.62
Gender (%)			0.99
Male	72 (66.7)	212 (66.7)	
Female	36 (33.3)	106 (33.3)	
GA (mean) (SD) *	29.9 (6.00)	38.4 (1.07)	<0.001
BBW (gm) (SD) *	1420 (591)	3053 (395)	<0.001
Stratified by age (yrs) (%)			0.86
0–1	30 (27.8)	93 (29.3)	
1–5	78 (72.2)	225 (70.7)	
Mean 1st FS duration (mins) (SD) *	8.69 (10.05)	5.81 (5.58)	0.03
No. of FS (SD)	1.44 (0.65)	1.35 (0.62)	0.47
Brain condition within 6 months of age (%)			<0.001
Negative	45 (41.6)	312	
IVH	45 (41.6)	-	
HCP or VM	6 (5.5)	6	
WM or PVL	12 (11.1)	-	
Others (HCC, microcephaly)	6 (5.5)	-	
Neurodevelopmental disabilities (%)			<0.001
Epilepsy	12 (11.1)	15 (4.71)	
ASD	15 (13.8)	3 (0.94)	
ADHD	30 (27.7)	18 (5.66)	

BBW, Birth body weight; GA, Gestational age; HCC, hypogenesis of the corpus callosum; HCP, *Hydrocephalus*; IVH, Intraventricular hemorrhage; mins, minutes; PVL, Periventricular leukomalacia; VM, ventriculomegaly; WM, White matter damage; yrs, years, * *t*-test.

**Table 2:** Incidence Rates and Odds Ratio of Epilepsy, Autism Spectrum Disorder and Attention Deficit Hyperactivity Disorder for the FS+/Preterm− and FS+/Preterm+ Groups and Those Stratified by Age and Number of Febrile Seizures Using a Logistic Regression Model.

Group	Epilepsy			ADHD			ASD		
	Event (No.)	IR (%, *N*)	OR (95% CI)	Event (No.)	IR (%, *N*)	OR (95% CI)	Event (No.)	IR (%, *N*)	OR (95% CI)
Term children with FS (*N* = 318)	15	4.71	Reference	18	5.66	Reference	3	0.94	Reference
Age (yrs, n)									
0–1 (93)	3	0.94	Reference	6	1.88	Reference	1	0.31	Reference
1–5 (225)	12	3.77	Reference	12	3.77	Reference	2	0.62	Reference
No of FS									
1 (225)	0	0	-	15	4.71	Reference	1	0.31	Reference
>1 (93)	15	4.71	Reference	3	0.94	Reference	2	0.62	Reference
Preterm children with FS (*N* = 108)	12	11.1	2.52 (1.14, 5.58) *	30	27	6.41 (3.39, 12.09) ***	15	13.9	16.9 (4.79, 59.7) ***
Age (yrs, n)									
0–1 (30)	3	2.77	3.33 (0.63, 17.4)	6	5.54	3.62 (1.07, 12.2) *	3	2.77	10.22 (1.02,102.3) *
1–5 (78)	9	8.33	2.31 (0.93, 5.72)	24	22.2	7.88 (3.70, 16.7) ***	12	11.1	20.27 (4.4,92.8) ***
No of FS									
1 (27)	3	2.77	-	15	13.9	17.5 (6.95, 44.0) ***	6	5.55	64.0 (7.35, 557) ***
>1 (81)	9	8.33	0.65 (0.26, 1.57)	15	13.9	6.81 (1.89, 24.5) **	9	8.33	5.68 (1.19, 27.1) *

IR, Incidence rate; OR, Odds ratio; CI, Confidence interval; ASD, Autism spectrum disorder; ADHD, Attention Deficit Hyperactivity Disorder, * *p* < 0.05, ** *p* < 0.01, *** *p* < 0.001.

**Table 3:** Odds Ratio of Autism Spectrum Disorder and Attention Deficit Hyperactivity Disorder for FS+/Preterm+ Children with Different Classifications and Their Baseline.

FS+/Preterm+ (*n* = 108)	ASD		ADHD	
	OR (95% CI)	*p*	OR (95% CI)	*p*
Sex ^ 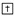 ^				
F	0	0.99	2.50 (0.43, 14.23)	0.30
GA (wks) ^ 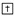 ^				
30–35	1.11 (0.07, 15.5)	0.9	0.37 (0.04, 2.99)	0.35
<30	0.58 (0.04, 7.91)	0.68	0.26 (0.03, 1.86)	0.18
Brain condition ^ 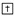 ^				
Abnormal	1.14 (0.16, 7.89)	0.88	0.67 (0.15, 2.91)	0.59
No. of FS ^ 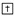 ^				
>1	3.15 (0.45, 21.94)	0.24	2.25 (0.5, 10.01)	0.28
1st FS duration (mins) ^ 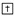 ^				
>10	1.04 (0.09,11.09)	0.97	0.37 (0.03, 3.54)	0.38
SGA ^ 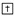 ^				
Yes	0	0.99	2.35 (0.42, 13.18)	0.33

FS: febrile seizure; GA: gestational age; SGA: Small for gestational age; wks: weeks. 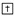 Reference for sex (male), GA (35–37 weeks); brain condition at birth (normal); number of FSs (1); first FS duration (1–10 min); SGA (No).

## Data Availability

Datasets are available on request.

## Abbreviations

ADHD	Attention Deficit Hyperactivity Disorder
ASD	Autism Spectrum Disorder
CI	Confidence Interval
DSM	Diagnostic and Statistical Manual of Mental Disorders
FS	Febrile Seizure
MR	Mental Retardation
NDDs	Neurodevelopmental Disorders
OR	Odds Ratio
TS	Tourette Syndrome
